# Photonic Reservoir Computer with Output Expansion for Unsupervized Parameter Drift Compensation

**DOI:** 10.3390/e23080955

**Published:** 2021-07-26

**Authors:** Jaël Pauwels, Guy Van der Sande, Guy Verschaffelt, Serge Massar

**Affiliations:** 1 Laboratoire d’Information Quantique, Université Libre de Bruxelles, B-1050 Bruxelles, Belgium; serge.massar@ulb.be; 2 Applied Physics Research Group, Vrije Universiteit Brussel, B-1050 Ixelles, Belgium; guy.van.der.sande@vub.be (G.V.d.S.); guy.verschaffelt@vub.be (G.V.)

**Keywords:** photonic computing, reservoir computing, coherent optical reservoir, output expansion, readout weight-tuning, unsupervised noise compensation

## Abstract

We present a method to improve the performance of a reservoir computer by keeping the reservoir fixed and increasing the number of output neurons. The additional neurons are nonlinear functions, typically chosen randomly, of the reservoir neurons. We demonstrate the interest of this expanded output layer on an experimental opto-electronic system subject to slow parameter drift which results in loss of performance. We can partially recover the lost performance by using the output layer expansion. The proposed scheme allows for a trade-off between performance gains and system complexity.

## 1. Introduction

Photonic reservoir computing is a neuro-inspired computing scheme which continues to push the boundary of optical computing towards faster and more energy efficient computing systems. Designed to exploit transient nonlinear dynamics to perform useful computation, the reservoir computing framework from [1,2,3] is applicable to a wide variety of physical systems. Photonic implementations tap into high bandwidth and versatile platforms, ranging from free-space optics to fiber-based systems and integrated optical circuits. Different flavors of electronic, opto-electronic and all-optical reservoirs exist, including delay-based systems [4,5,6,7,8,9,10,11,12], network-based systems [13,14,15,16,17] and speckle-based systems [18,19,20,21]; an overview is given in [22]. Noise and parameter variations can affect a reservoir’s performance in different ways, as was investigated, for example, in [23,24,25].

To solve computational tasks, reservoir outputs are constructed, which encode the reservoir’s best approximation to the solution of the computational task. Reservoir outputs are typically constructed as linear combinations of the reservoir’s output features. Typically, the reservoir’s output features correspond directly with the signals provided by the reservoir’s neurons, and the weights in the linear combinations are called the readout weights. If the desired output signals are known, linear regression allows the readout weights to be optimized with little effort.

In this work, we present a method to increase the number of output features to improve a reservoir’s performance. The reservoir itself remains unchanged with random and fixed connections between the neurons. Additional output features are obtained as nonlinear functions of the original reservoir neurons. In the spirit of reservoir computing, these functions can be random. We show that even with additional output neurons, the standard training procedure remains applicable. The extended set of output neurons simply means that an extended set of readout weights has to be optimized with the same standard methods used for reservoir computers. The proposed scheme allows one to expand the computational power of a reservoir computer without expanding the recurrent part of the reservoir itself. This allows a trade-off which may be interesting for experimental implementations.

We demonstrate the interest of this expanded output layer on an experimental photonic reservoir computing system, following [12], subject to slow parameter drift (i.e., slow with respect to the timescale of the task-related input data). These unintentional variations could, for example, be caused by drifts in ambient parameters (such as temperature) and result in loss of performance. This is because the optimization of the readout weights, which is based on simple linear regression, can depend strongly on all parameters that affect the reservoir’s operation. If a reservoir is trained for different but fixed sets of operational parameters, the resulting optimized readout weights generally differ. Using any of these optimized sets of weights leads to a suboptimal approximation to the task solution if the operational parameters are continuously drifting during operation. With the proposed output expansion scheme, we can partially recover the performance lost due to parameter fluctuations. We note that the employed scheme is expected to improve the reservoir’s performance regardless of the presence of parameter drifts. Our focus, however, lies in performance recovery under the presence of such drifts.

The problem of compensating for parameter drift was investigated previously in [25]. There, a simulated coherent photonic reservoir computer was considered, with slow variations to the roundtrip phase of the optical cavity that makes up the photonic reservoir. Auxiliary reservoir outputs were trained to recover these cavity phase variations and were used to tune the task-related output weights. As will be discussed, this weight-tuning scheme is in fact equivalent to (and thus an example of) a fixed reservoir with expanded output layer. This adapted reservoir output scheme was shown to improve robustness to phase fluctuations. A supervised learning scheme was employed to extract the drifting parameter from the neural responses, limiting the approach to drift compensation for parameters which can be readily measured and which entail a corresponding experimental overhead. We adapt and experimentally verify this method as we switch to an unsupervised method, as described above, to compensate for the drifting parameters in a physical system. The advantage of an unsupervised method is that it does not require the drifting parameters to be measured, estimated or even identified.

Firstly, in Section 2 of this paper, we explain the standard mode of operation of a reservoir computer and the principle of output layer expansion. We then discuss how dynamic parameter variations are expected to affect the system performance. We subsequently summarize previous efforts to mitigate performance losses due to parameter fluctuations and explain—in more detail—our solution, which is based on the expansion of the reservoir’s output layer. The experimental setup that we used to validate the proposed approach is then presented in detail. Next, in Section 3, we show our analysis of the information content of the additional reservoir outputs we constructed. Then, we show how the computational capacity of the system is improved by the proposed scheme, and we present the benchmark performance of our adapted reservoir computer on a four-level channel equalization task. Finally, the impact of these findings is discussed, and concluding remarks are presented in Section 4.

## 2. Materials and Methods

### 2.1. Reservoir Computing with Output Layer Expansion

In this section, we cover the reservoir computing basics in terms of operation and training and discuss the principle of output layer expansion. A reservoir computer consists of *N* internal states, also called neurons, here captured in a column vector X(n) as a function of discrete time *n*. The system is operated by coupling input data I(n) to these neurons using different weights. The neurons are randomly interconnected to form a recurrent neural network. A state update equation describes how the neural states evolve, i.e., how their (typically nonlinear) activation function $f_{act}$ acts (element-wise) on both past states and newly injected data as

(1) $$\begin{array}{c} X\left(n+1\right)=f_{act}\left(X(n),I(n)\right). \end{array}$$

Standard reservoir outputs are constructed through linear combinations of neural responses with a set of readout weights $W_{out}$. In practice, the act of accessing these neural responses often involves measuring and recording them. The measured responses $X_{m}$, with subscript *m*, are then obtained by parsing the neural responses with a (possibly nonlinear) readout function $f_{m}$ acting element-wise; we note $X_{m}=f_{m}\left(X\right)$. In our work, we will be measuring the optical power of the neural states encoded in the optical field strength, such that $X_{m}={\left|X\right|}^{2}$. For a standard reservoir output, then, the measured responses form the set of output features $X_{out}$ that are combined with output weights $W_{out}$ to construct reservoir outputs:

(2) $$\begin{array}{c} X_{out}=X_{m}={\left|X\right|}^{2}. \end{array}$$

Below, we will generalize Equation (2) to a scenario where the output features $X_{out}$ are not restricted to equal the measured responses $X_{m}$. The number of output features is denoted $N^{\prime }$ (in the case of Equation (2) we have $N^{\prime }=N$).

Multiple parallel reservoir outputs can be created from these output features. Since all outputs are created in the same way, we focus here on a single scalar output $Y_{out}$. This output is constructed by optimizing a row vector of $N^{\prime }$ readout weights $W_{out}$:

(3) $$\begin{array}{c} Y_{out}\left(n\right)=W_{out}X_{out}\left(n\right). \end{array}$$

By combining *T* timesteps, we construct row vector $Y_{out}$ with $Y_{out}\left(n\right)$ as its $n^{th}$ element and state matrix $X_{out}$ with $X_{out}\left(n\right)$ as its $n^{th}$ column to rewrite Equation (3) as

(4) $$\begin{array}{c} Y_{out}=W_{out}X_{out}. \end{array}$$

The readout weights $W_{out}$ are optimized to minimize the square error with respect to a target output $Y_{target}$ by finding the pseudo inverse $X_{out}^{†}$ of the state matrix $X_{out}$ as

(5) $$\begin{array}{c} W_{out}=\underset{W}{\mathrm{argmin}}{∥Y_{target}-WX_{out}∥}_{2}^{2}=Y_{target}X_{out}^{†}. \end{array}$$

Typically, the readout weights are optimized over a set of training samples, and the residual output error is evaluated over a disjunct set of testing samples.

In this work, we consider an adapted readout layer which expands the number of output features $X_{out}$ using polynomials of the measured states $X_{m}$. In general, such an adaptation to the readout layer can be described by an output function:

(6) $$\begin{array}{c} f_{out}:R^{N}\to R^{N^{\prime }}:X_{m}\left(n\right)\mapsto X_{out}\left(n\right)=f_{out}\left(X_{m}\left(n\right)\right) \end{array}$$

which maps the *N* dimensional vector $X_{m}\left(n\right)$ of (measured) neural responses to an $N^{\prime }$ dimensional vector of output features $X_{out}\left(n\right)=f_{out}\left(X_{m}\left(n\right)\right)$. Any readout adaptation of this form has the advantage that the standard procedure for constructing and training reservoir outputs given by Equations (3)–(5) remains applicable.

### 2.2. Output Expansion with First and Second Degree Polynomials

Here, we present an illustrative example of an output feature expansion. This example will be used below to improve the performance of an experimental photonic reservoir computing system. It is important, however, to keep in mind that the proposed scheme can be generalized in many ways (there are many other nonlinear expansions possible). With this example expansion, we pay special attention to the number of output features. This is because it allows us to probe the trade-off between system complexity and computational performance, a trade-off which is of great interest to experimental reservoir computing systems in general.

We have chosen to expand the reservoir’s set of output features with polynomial functions, limited to first and second degree, of the recorded neural responses. The first degree contributions correspond with the original recorded responses $X_{m}$. The second degree contributions are obtained by mixing the recorded responses $X_{m}$ with auxiliary features. These auxiliary features are signals constructed as linear combinations of the recorded responses $X_{m}$. This process is identical for standard reservoir outputs, which is why we label these auxiliary features as *Y*, and we will add a subscript to refer to the method used to obtain them. In general, the auxiliary features can be constructed through supervised training or unsupervised training, determined by the availability of a target signal. Here we focus on an unsupervised method to obtain the auxiliary features because we want to avoid the need to measure, estimate or even identify all drifting parameters, and consequently, no target signals are available.

Since parameter drifts and variations are expected to occur slowly with respect to the input sample spacing, we have tried using slow feature analysis [26] to find linear combinations of the neural responses that vary slowly. Our efforts are outlined in Appendix B. This method yields useful slow features, i.e., signals that correlate strongly with the slowest perturbations of the system, which are the parameter drifts. However, we also found that constructing random auxiliary features $Y_{R}$ as linear combinations of the recorded neural responses $X_{m}$ with random weights $W_{R}$ is much easier than constructing slow features and gives the same performance gains and robustness to parameter drifts. For this reason, we focus on the latter approach.

In general the practical implementation determines the bounds of the distribution from which the random weights are sampled. Since we evaluate the proposed scheme by post-processing the recorded experimental data, we have complete liberty. Here, we choose to sample weights uniformly from [−1,1] as this could be implemented passively (i.e., without amplification).

(7) $$\begin{array}{c} W_{R}∼\mathrm{Uniform}\left[-1,+1\right]. \end{array}$$

To obtain *P* random features, the P×1 column vector $Y_{R}\left(n\right)$ is thus constructed with the P×N matrix $W_{R}$ as

(8) $$\begin{array}{c} Y_{R}\left(n\right)=W_{R}X_{m}\left(n\right). \end{array}$$

We now write the explicit form of the corresponding output expansion $f_{out}$ to clarify how we obtain the full set of output features $X_{out}$. Combining *T* timesteps, we construct the $N^{\prime }\times T$ matrix of output features $X_{out}$ with $N^{\prime }=N\left(P+1\right)$ where the $n^{th}$ column $X_{out}\left(n\right)$ is constructed as follows: the first *N* elements correspond with the measured neural responses $X_{m}\left(n\right)$, the next *N* elements correspond with $X_{m}\left(t\right)$ multiplied by the first element of $Y_{R}\left(n\right)$, the next *N* elements are $X_{m}\left(t\right)$ multiplied by the second element of $Y_{R}\left(n\right)$ and so on. Using the Kronecker product, this can be written as

(9) $$\begin{array}{cc} X_{out}\left(n\right) & =\left[\begin{array}{c} X_{m}\left(n\right)\\ Y_{R}^{⊤}\left(n\right)⊗X_{m}\left(n\right) \end{array}\right]. \end{array}$$

The output features thus consist of the recorded neural responses directly and these responses mixed with the random features. The extended set of $N^{\prime }=N\left(P+1\right)$ readout weights $W_{out}$ can then be obtained following the standard training procedure of Equation (5). Note that P≤N, as otherwise there will be output features $X_{out}$ which are linearly dependent on other output features.

The relation between the task-solving reservoir output $Y_{out}$ and the expanded set of output features $X_{out}$ is still given by Equation (3). Additionally, for the specific output expansion discussed here, we can express $Y_{out}$ in terms of the recorded neural responses $X_{m}$ directly as

(10) $$\begin{array}{c} Y_{out}\left(n\right)=W_{out}^{\left(1\right)}X_{m}\left(n\right)+X_{m}^{⊤}\left(n\right)W_{R}^{⊤}W_{out}^{\left(2\right)}X_{m}\left(n\right). \end{array}$$

where the $W_{out}^{\left(1\right)}$ is the subset of readout weights in $W_{out}$ used for first order polynomials terms in $X_{m}$, and the matrix product $W_{R}^{⊤}W_{out}^{\left(2\right)}$ is the subset used for second order terms. More formally, vectorizing weights matrix $W_{out}^{\left(2\right)}$ and appending it to weights vector $W_{out}^{\left(1\right)}$ yields $W_{out}$ as

(11) $$\begin{array}{c} W_{out}=\left[W_{out}^{\left(1\right)}\;\;\mathrm{vec}{\left(W_{out}^{\left(2\right)}{\;}^{⊤}\right)}^{⊤}\right]. \end{array}$$

**Figure 1:**
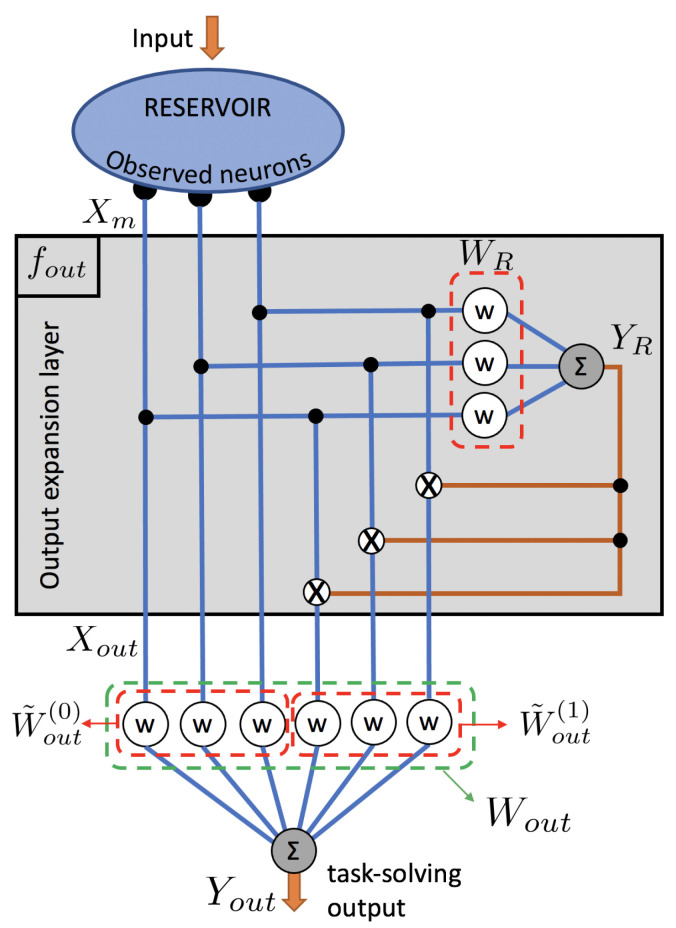
Illustration of the output expansion scheme. In the example shown, 3 neurons are measured $X_{m}$, 1 random feature $Y_{R}$ is constructed with random weights $W_{R}$ and this auxiliary feature is mixed with $X_{m}$ to obtain a total of 6 output features $X_{out}$. These output features are combined with trained readout weights $W_{out}$ to form a task-solving reservoir output $Y_{out}$. This output expansion contains polynomial functions of $X_{m}$ of first and second degree. The corresponding subsets of readout weights are labeled $W_{out}^{\left(1\right)}$ and $W_{out}^{\left(2\right)}$. Larger numbers of neurons N and auxiliary features P are supported by the proposed scheme.

Note that the N×N matrix resulting from the product of $W_{R}^{⊤}$ and $W_{out}^{\left(2\right)}$ is not necessarily of full rank, depending on the (number of) features used. In an explicit nonlinear expansion of this degree, this matrix product would be replaced by a single N×N matrix of full rank. Our approach allows for choosing how many auxiliary features are used, which offers a trade-off between performance gains and system complexity, as will be shown in the Results section. This output-expansion scheme is illustrated in Figure 1, showing 3 neural responses and 1 random feature resulting in 6 output features.

### 2.3. Slow Noise and Feature Dependent Weights

In this section, we discuss slow uncontrolled parameter variations which can affect the internal dynamics of a reservoir computer in operation and can thus negatively impact its performance. We also touch on the concept of feature-dependent weights and how it relates to the previously discussed nonlinear output expansion.

Denote by θ the set all parameters and operators describing a reservoir computer’s operation in Equation (1). Following the spirit of reservoir computing, we avoid micromanaging the reservoir’s response to changes in θ and instead focus on the optimization of the readout weights $W_{out}$. If θ changes over time due to environmental fluctuations (which are slow with regard to the input data rate), then the system’s computational capacity can be negatively affected. The standard reservoir training scheme will automatically try to capture the reservoir’s dynamics for the range of values θ encountered during training. This provides a natural robustness during testing, provided that only similar values of θ occur. However, this robustness obviously comes at a price, since a reservoir trained and tested on a fixed parameter set θ would work better. Furthermore, even without parameter drifts or variations, reservoir computing systems can exhibit different performance levels at different operating points. So even if the system is, in principle, not affected by the dynamics of the parameter fluctuations, performance variations could still occur due to the suitability of the instantaneous parameter values.

In [25], several approaches are presented to counter the negative impact of such variations on the performance of a photonic reservoir computer. There, a simulated coherent reservoir is perturbed by the variations of a single parameter θ (one-dimensional), namely the detuning of an optical cavity. An estimation of any uncontrolled variations in θ is extracted from the reservoir using 2 auxiliary features, $Y_{\cos\theta }$ and $Y_{\sin\theta }$. As their names suggest, these features are trained to estimate cosθ and sinθ to account for the periodic nature of the system’s response to changes in θ. These two auxiliary features are constructed as standard reservoir outputs, i.e., as linear combinations of the neural responses with readout weights $W_{\cos\theta }$ and $W_{sin\theta }$, respectively. These weights are obtained through the regular (supervised) reservoir training procedure, Equation (5), since the target signals (cosθ and sinθ) are known. These features are obtained as

(12) $$\begin{array}{c} Y_{\cos\theta }\left(n\right)=W_{cos\theta }X_{m}\left(n\right) \end{array}$$

(13) $$\begin{array}{c} Y_{\sin\theta }\left(n\right)=W_{sin\theta }X_{m}\left(n\right) \end{array}$$

and thus, omitting the additional filtering that was applied in [25] to clean up these auxiliary features, this constitutes an example of auxiliary features such as presented previously, albeit with non-random weights.

Furthermore, in [25], recognizing that fixed readout weights yield suboptimal solutions under varying θ, a weight-tuning scheme is then implemented, changing the output relation Equation (3) to

(14) $$\begin{array}{cc} Y_{out}\left(n\right) & ={\tilde{W}}_{out}\left(n\right)X_{m}\left(n\right) \end{array}$$

with time-dependent readout weights

(15) $$\begin{array}{c} {\tilde{W}}_{out}\left(n\right)=W_{out}^{\left(1\right)}+Y_{\cos\theta }\left(n\right)W^{\left(c\right)}+Y_{\sin\theta }\left(n\right)W^{\left(s\right)}. \end{array}$$

This results in an extended set of weights $\left({\tilde{W}}_{out}^{\left(1\right)},{\tilde{W}}^{\left(c\right)},{\tilde{W}}^{\left(s\right)}\right)$ that is optimized during supervised training, using the target output specified for the computational task at hand. It has successfully been shown that this weight-tuning scheme improves the robustness to phase fluctuations of the simulated photonic reservoir computer and provides good performance over a wide range of operational settings. In fact, it does so without taxing the reservoir’s computational capacity as it is no longer the reservoir’s internal dynamics which provide the robustness to parameter variations, but rather the readout weight-tuning.

We have identified this weight-tuning scheme to be an alternative perspective on the nonlinear output expansion with polynomials of first and second degree, as discussed above, a perspective which is also useful for the optimization of the extended set of readout weights, as discussed in the next paragraph. In this work we effectively build on this idea, switching to an unsupervised method (in the form of random features as discussed above) to demonstrate the concept experimentally. We consider a different coherent photonic reservoir computer but with the same one-dimensional θ, i.e., the detuning of the optical cavity that makes up the reservoir. This system allows us to compare simulation results directly with experiments. We employ the same readout layer adaptation, but instead of constructing estimates of cosθ and sinθ through supervised training, we explore the applicability of the proposed adapted readout scheme to drifting parameters which cannot readily be measured. Lacking (and preventing the need for) a measurement of θ, we do not train the additional outputs with the standard procedure Equation (5). Instead, as discussed above, we construct random features following Equation (8).

The specific example of a nonlinear output expansion that we presented is, in fact, equivalent to the same weight-tuning scheme presented in [25]. In our case, the weight-tuning scheme is expressed as

(16) $$\begin{array}{c} {\tilde{W}}_{out}\left(n\right)=W_{out}^{\left(1\right)}+Y_{R}\left(n\right)W_{out}^{\left(2\right)} \end{array}$$

which, when combined with the output relation Equation (14), yields

(17) $$\begin{array}{cc} Y_{out}\left(n\right) & =\left(W_{out}^{\left(1\right)}+Y_{R}{\left(n\right)}^{⊤}W_{out}^{\left(2\right)}\right)X_{m}\left(n\right). \end{array}$$

**Figure 2:**
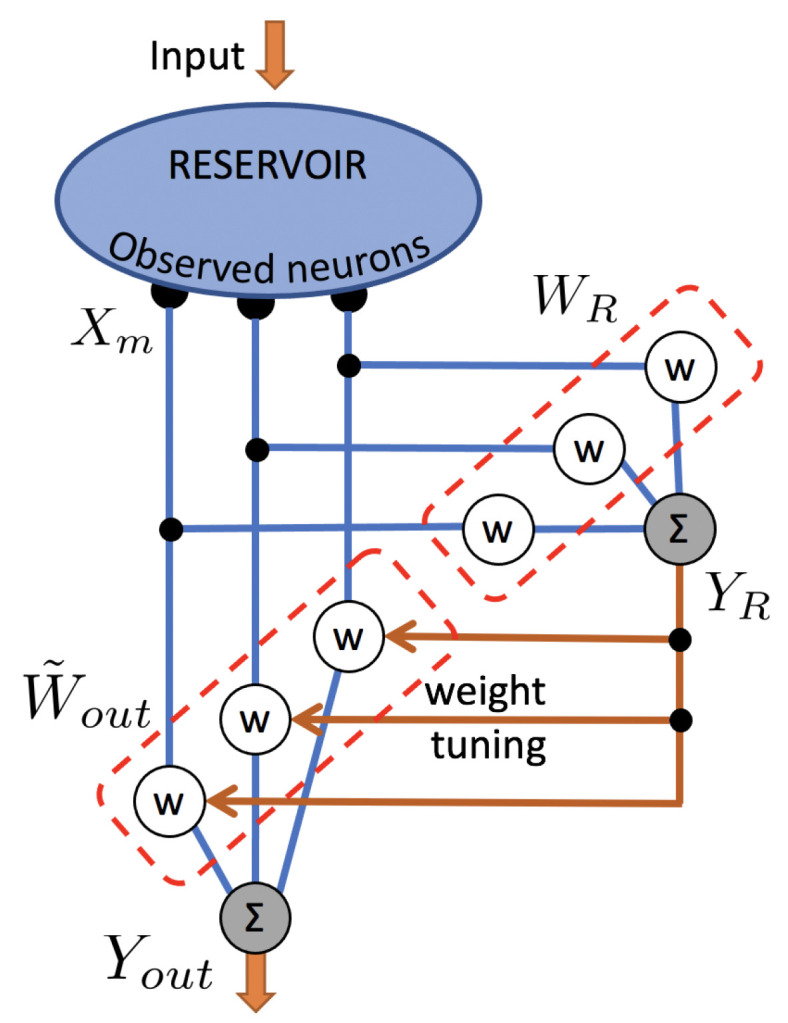
Illustration of the weight-tuning scheme. The example shows 3 measured neural responses $X_{m}$ which are combined with 3 time-dependent readout weights ${\tilde{W}}_{out}$ following Equation (16) to form 1 task-solving output $Y_{out}$ following Equation (17). The example has 1 random auxiliary feature $Y_{R}$, which is obtained with random weights $W_{R}$ and used to tune ${\tilde{W}}_{out}$. Larger numbers of neurons *N* and auxiliary features *P* are supported by the proposed scheme. This example is equivalent with the scheme shown in Figure 1.

One can verify that Equations (8) and (17) indeed combine to yield Equation (10), which confirms the equivalence. The weight-tuning scheme is illustrated in Figure 2, showing 3 measured neural responses $X_{m}$ which are combined with 3 time-dependent readout weights ${\tilde{W}}_{out}$ to form 1 task-solving output $Y_{out}$. It also shows 1 auxiliary random feature $Y_{R}$ which is obtained with random weights $W_{R}$ and used to tune the time-dependent readout weights ${\tilde{W}}_{out}$, and it can be compared with Figure 1 to verify that it is an example of a nonlinear output expansion.

### 2.4. Setup

**Figure 3:**
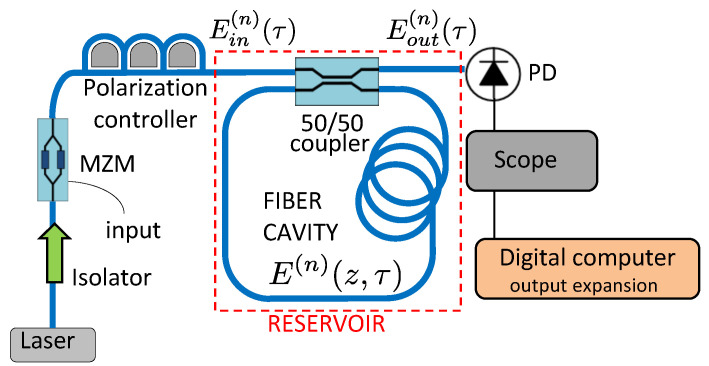
Schematic of the fiber-ring cavity of length *L* used to implement an optical reservoir. In the input layer, a polarization controller maps the input polarization onto a polarization eigenmode of the cavity. Data is injected by means of a Mach–Zehnder modulator (MZM). A coupler with power transmission coefficient T=50% couples the input field $E_{in}^{\left(n\right)}\left(\tau \right)$ to the cavity field $E^{\left(n\right)}\left(z,\tau \right)$ and couples to the output field $E_{out}^{\left(n\right)}\left(\tau \right)$, where *n* is the roundtrip index, τ is time (with $0<\tau <t_{R}$) and *z* is the longitudinal position in the ring cavity. A photodetector (PD) records the neural responses to be processed by a digital computer where the output expansion is realized.

In this section we discuss the dynamical system on which our reservoir computing simulations and experiments is based. The reservoir itself is implemented in the all-optical fiber-ring cavity shown in Figure 3, using standard single-mode fiber. A polarization controller is used to ensure that the input field $E_{in}$ excites a polarization eigenmode of the fiber-ring cavity. A fiber coupler, characterized by its power transmission coefficient T=50%, couples light in and out of the cavity. Ignoring dispersion, the fiber-ring is characterized by the roundtrip length L=10 m (or roundtrip time $t_{R}=50$ ns), the propagation loss α (taken here 0.18 dBk$m^{-1}$), the fiber nonlinear coefficient $\gamma_{Kerr}=2.6\;\mathrm{mrad}m^{-1}W^{-1}$ and the cavity detuning θ, i.e., the difference between the roundtrip phase and the nearest resonance (multiple of 2π). Without active stabilization, the cavity detuning is an uncontrolled parameter susceptible to slow (sub-MHz) variations. This low-finesse cavity is operated off-resonance, with a maximal input power of 50 mW (17 dBm). A network of time-multiplexed virtual neurons is encoded in the cavity field envelope, with neuron spacing Δτ.

We use the physical model constructed in [12]. In this mean-field model, the temporal evolution of the electric field envelope is described by $E^{\left(n\right)}\left(z,\tau \right)$, which represents the cavity field envelope measured at position *z* from the coupler at time τ during the *n*-th roundtrip. The longitudinal coordinate of the fiber ring cavity is bound by the cavity length 0<z<L, and similarly, the time variable is bound by the cavity roundtrip time $0<\tau <t_{R}$, since other values are covered by the expressions of other roundtrips (with different *n*). A nonlinear propagation model is combined with the cavity boundary conditions to transform the input field $E_{in}^{\left(n\right)}\left(\tau \right)$ into the output field $E_{out}^{\left(n\right)}\left(\tau \right)$, following the equations

(18) $$\begin{array}{cc} E^{\left(n\right)}\left(L,\tau \right) & =E^{\left(n\right)}\left(0,\tau \right)\exp\left(i\gamma |E^{\left(n\right)}{\left(0,\tau \right)|}^{2}L_{eff}-\alpha L\right) \end{array}$$

(19) $$\begin{array}{cc} E^{\left(n+1\right)}\left(0,\tau \right) & =\sqrt{T}E_{in}^{\left(n+1\right)}\left(\tau \right)+\sqrt{1-T}e^{i\theta }E^{\left(n\right)}\left(L,\tau \right) \end{array}$$

(20) $$\begin{array}{cc} E_{out}^{\left(n+1\right)}\left(\tau \right) & =\sqrt{1-T}E_{in}^{\left(n+1\right)}\left(\tau \right)+\sqrt{T}e^{i\theta }E^{\left(n\right)}\left(L,\tau \right) \end{array}$$

where the effective cavity length that describes the accumulation of nonlinear Kerr phase is $L_{eff}={\left(2\alpha \right)}^{-1}\left(1-exp\left(-2\alpha L\right)\right)$. In this model, the cavity phase is the only drifting/noisy parameter and is therefore denoted θ(n). Variations in θ are caused by drift in the frequency of the pump laser and mechanical/thermal fluctuations affecting the cavity.

The input field $E_{in}$ is generated by using a Mach–Zehnder modulator (MZM) to modulate a CW optical pump following [7]. Here the input signal u(n) (∈[−1,1]) is first mixed with the input masks $b_{k}$ and $m_{k}$ (with neuron index *k*):

(21) $$\begin{array}{c} {\tilde{u}}_{k}\left(n\right)=b_{k}+m_{k}u\left(n\right) \end{array}$$

and then used to drive the MZM. The input coupled to the *k*-th neuron is thus expressed as

(22) $$\begin{array}{c} I_{k}\left(n\right)=\sqrt{P_{0}}\;\cos\left(\beta_{0}+\beta_{1}{\tilde{u}}_{k}\left(n\right)\right) \end{array}$$

where $P_{0}$ represents the pump power, $\beta_{0}\approx \pi /2$ represents the setpoint and $\beta_{1}\approx \pi /4$ represents the modulation range. The bias mask values $b_{k}$ allow the masked input values ${\tilde{u}}_{k}\left(n\right)$ to exploit the full modulation range and affect the reservoir’s ability to recover phase information from its neural responses. These inputs then couple to all neurons through time-multiplexing, as the amplitude-modulated input field becomes

(23) $$\begin{array}{c} E_{in}^{\left(n\right)}\left(\left(k-1\right)\Delta \tau <\tau <k\Delta \tau \right)=I_{k}\left(n\right). \end{array}$$

The neuron spacing Δτ is set with respect to the cavity roundtrip time $t_{R}$ and the number of neurons *N* as

(24) $$\begin{array}{c} \Delta \tau =\frac{t_{R}}{N+1}. \end{array}$$

This deviation from the synchronized scenario ($\Delta \tau =t_{R}/N$) yields a ring-like coupling topology between the neurons, following [9].

The output field $E_{out}$ is sent to the readout layer where the neural responses are demultiplexed. In the readout layer, a photodetector (PD) measures the optical power of the neural responses $|E_{out}{|}^{2}$. More specifically, the measured value of the *k*-th neuron is

(25) $$\begin{array}{c} X_{m,k}\left(n\right)=\frac{1}{\Delta \tau }\int_{nt_{R}+\left(k-1\right)\Delta \tau }^{nt_{R}+k\Delta \tau }{\left|E_{out}^{\left(n\right)}\left(\tau \right)\right|}^{2}d\tau . \end{array}$$

The expansion of the reservoir’s output layer will be achieved by digitally post-processing the experimentally recorded neural responses.

It is known that the MZM and PD can act nonlinearly on the input and output signals and can thus affect the RC system’s performance [9]. The implications for a coherent nonlinear reservoir have been investigated in [12].

With high optical power levels and small neuron spacing (meaning fast modulation of the input signal), dynamical and nonlinear effects other than the Kerr nonlinearity may appear, such as photon–phonon interactions causing Brillouin and Raman scattering and bandwidth limitations caused by the driving and readout equipment. These effects are not included in our numerical model, and our experiments are designed to avoid them. Combined with the memory limitations of the oscilloscope, we therefore limit our reservoir to 20 neurons, with a maximal input power of 50 mW.

We remark a particular symmetry to this system. The Equations (18)–(20) admit a solution of the form $E_{out}^{\left(n\right)}\left(\tau \right)=F\left(\gamma ,L,\theta ,E_{in}^{\left(n\right)}\left(\tau \right),E_{in}^{\left(n-1\right)}\left(\tau \right),\cdots \right)$ for some function *F* which can be computed iteratively. With a real-valued input signal $E_{in}$, the complex conjugate of $E_{out}$ is given by the same function, with opposite signs for θ and γ. Thus, neglecting the relatively weak influence of γ and following Equation (25), the recorded neural responses $X_{m}$ are an even function of θ.

In Appendix A, we consider a discrete time version of a linearized system model, and we investigate how θ(n) affects the recorded neural responses (and linear combinations thereof, such as our random features). When averaged over times which are long compared to the cavity roundtrip time $t_{R}$ but short compared to the time over which θ(n) varies and when the input bias $b_{k}$ is non-zero, the system response depends on $\cos{\langle \theta \rangle }_{n}$ and its powers. These are even functions of θ, as expected on account of the mentioned symmetry.

## 3. Results

### 3.1. Ability of Random Features to Capture Parameter Variations

In the envisioned scenario, we deal with losses in RC performance due to ambient parameter fluctuations. To understand how random auxiliary features can recover and boost performance when used to tune task-related readout weights, it is important to investigate what information is contained within these features. Hence, in this section we will use the setup outlined in Section 2.4 to investigate whether the random features correlate with the variations in the cavity roundtrip phase θ or detuning, which constitutes the uncontrolled parameter. To investigate whether the random features contain useful information about θ, we calculate the correlations between these features and cosθ.

**Figure 4:**
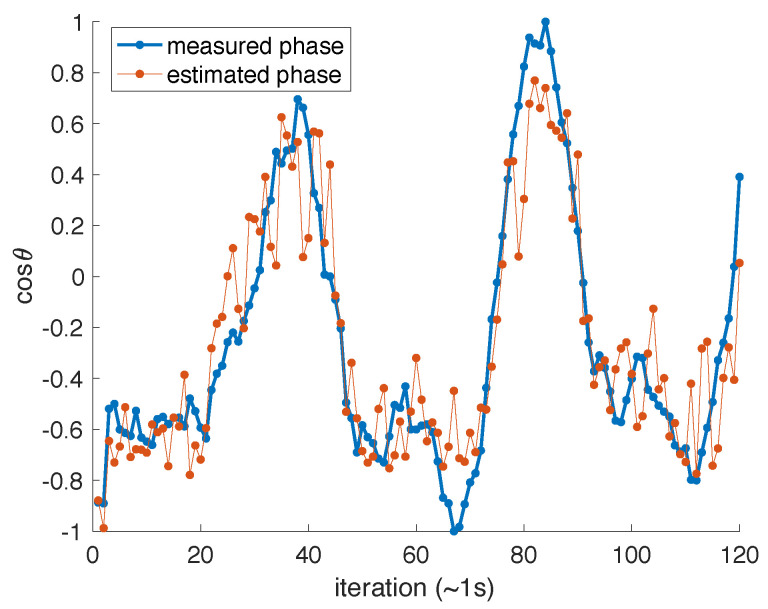
Example of experimental phase variations over different iterations of the experiment. The solid line is the measured phase, based on the pulse interference, and the dots represent the estimated phase using a linear combination of all 20 random features. The iterations take place approximately every second. The experiment is carefully shielded so that θ varies slowly.

In the experiment, we do not have direct access to a measure of the cavity phase. To obtain an estimate, we performed several iterations of the experiment, and the input data in each iteration were preceded by a series of well-timed pulses. We then analyze the interference between pulses that reflect off the optical cavity and pulses which exit the cavity after a roundtrip within the cavity. This scheme allowed us to periodically probe the cavity roundtrip phase between iterations of the experiment. More specifically, we recover an experimental estimate of cosθ every ∼1 s. In Figure 4, we show the estimated phase variations which are slow (sub-Hz), owing to our efforts to shield the setup from mechanical vibrations and temperature variations during these experiments. We also show the best approximation using a linear combination of all random features $Y_{R}$, obtained through linear regression following Equations (2), (4) and (5), which is seen to correctly capture the slow trends.

**Figure 5:**
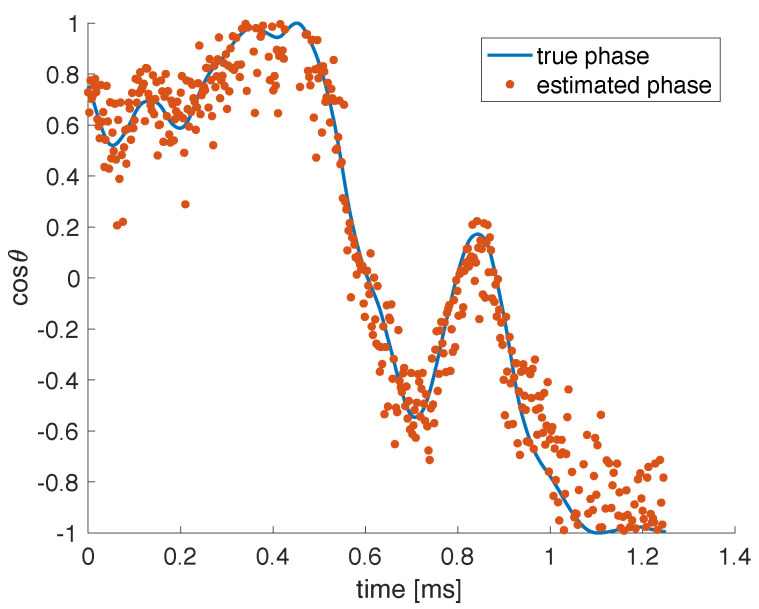
Example of simulated phase variations within 1 iteration of the simulated experiment. The full line represents the true phase variations, covering the full 2π range and with kHz bandwidth, and the dots represent the estimated phase, obtained using a linear combination of all 20 random features.

We also want to explore whether our approach can deal with faster phase fluctuations (up to ∼kHz). Thus, in further experiments we make no efforts to shield the setup from mechanical vibrations and temperature variations, which will allow us to investigate the system’s robustness to parameter changes within each experimental run. For this reason, we purposely perturb our simulations with random phase variations of the full 2π range and kHz bandwidth, to allow for significant variations within individual experiments, which last ∼1 ms. In Figure 5, we show an example of the simulated phase variations. We also show the best approximation using a linear combination of all random features. This approximation captures the correct slow trends, but fast variations at the timescale of the input data can also be observed. Indeed, from the point of view of measuring cosθ, the input signal is unwanted noise.

To determine how many random features are needed to obtain reliable information about θ, we try to reconstruct the phase variations with linear combinations of the *P* random features. The reservoir encodes N=20 neurons, and the random features are constructed as linear combinations of the (measured) neural responses. We therefore limit the number *P* of random features to 20. Additional features are not expected to contain any new information.

**Figure 6:**
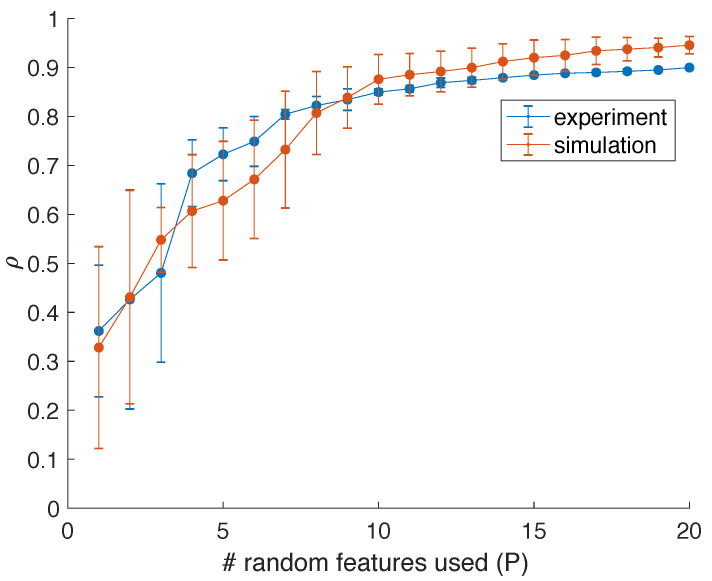
Correlation coefficients obtained when mapping increasing sets of random features to cosθ using linear regression. For the experimental comparison, an estimate of cosθ is used, whereas in simulation, the known value of cosθ is used. Error bars are obtained by running experiments/simulations for several iterations and using different sets of random weights for the construction of the random features.

We have quantified the accuracy in reconstructing cosθ when using an increasing number of features (*P*). In Figure 6, we show the Pearson correlation coefficient between the experimental estimates or simulated values of cosθ and the best approximation using subsets of the extracted features. The error bars are obtained by averaging the results over multiple sets of random features. Both in the experiment and simulation, at least four features are needed to obtain consistent correlation results above 50%, and correlation coefficients up to 90% can be reached when using more features.

### 3.2. Memory Capacity

In this section, we evaluate how the adapted reservoir readout layer outlined in Section 2.2 improves the reservoir’s computational capacity or memory capacity when exploiting random features. The evaluation framework used here allows the system’s total information processing capacity to be quantified and is based on the reservoir’s capacity to reconstruct a large set of polynomial functions, following [27,28]. For this experiment, the input data consists of random samples drawn from a uniform distribution over the interval [−1,1]. The reservoir computer is then trained to reconstruct both linear and nonlinear polynomial functions of past inputs.

The polynomial functions are constructed by combining Legendre polynomials, which are orthogonal over the distribution of the input data and, as such, yield independent pieces of information about the system’s total memory capacity. The reservoir’s ability to reconstruct each function is evaluated by comparing the trained output *y* with the target $\hat{y}$ for previously unseen input samples. This yields a memory capacity *C* between 0 and 1, as

(26) $$\begin{array}{c} C=1-\frac{{\langle {\left(\hat{y}-y\right)}^{2}\rangle }_{t}}{{\langle {\hat{y}}^{2}\rangle }_{t}}. \end{array}$$

These capacities are typically grouped by the degree of the corresponding polynomial functions. After summing over all memory capacities of an equal degree, we can quantify the contributions of individual degrees to the total memory capacity of the RC, which is the sum over all degrees.

By this evaluation scheme, we gain insight into the reservoir’s linear memory capacity, i.e., the ability to retain past input samples, and its nonlinear memory capacity, i.e., the ability to apply nonlinear transformations to the retained information. By combining both linear and nonlinear memory capacities, we find the total memory capacity. With fading capacities of increasing degree, this total capacity has an upper bound given by the number of dynamical variables in the system: here, the number of virtual neurons. In practice, the total memory capacity is degraded by the presence of readout noise. Furthermore, there is a trade-off between linear and nonlinear memory capacity, depending on the operating regime of the dynamical system. In our system, the linear memory capacity corresponds with degrees 1 and 2 due to the photodetector in the reservoir’s readout layer, which effectively performs a squaring operation when measuring optical power levels instead of optical field amplitudes [12]. The nonlinear memory capacity is captured by higher degrees.

When evaluating memory capacities on finite data sets, there is a risk of overestimating the capacities *C*, whose estimator Equation (26) is plagued by a positive bias. Following [28], we employ a cutoff capacity $C_{co}\approx 0.02$ for 5000 test samples, and we discard capacity estimates below this cutoff.

We have investigated and present here the effects of the expanded output layer on the system’s computational capacity. The goal is not to achieve state-of-the-art performance, but rather to demonstrate that the performance can be improved through this output expansion. In our particular example of such an expansion, we exploit random combinations of neural responses, which we have shown in Section 3.1 to contain information about the uncontrolled variations in the cavity phase. We apply the standard reservoir computing training approach to the expanded set of output features, as we project them onto the target solution following Equation (5). We expect to achieve a higher accuracy, i.e., larger memory capacities, as we increase the number of random features used for the reservoir’s output expansion.

We have performed simulations with the stable cavity phase (not shown), which have yielded total memory capacities between 16 and 150 as we vary the number of features used from 0 to 20. As expected, without phase noise and with the standard reservoir output layer (0 features used), the obtained total memory capacity of 16 is close to the theoretical maximum given by the number of observed independent variables [28]: here, the number of neurons N=20. The expanded sets of output features now contain additional independent output variables. With N=20 independent neural responses $X_{m}$, the number of independent second order monomials in $X_{m}$ is N(N+1)/2=210, for a total of up to 230 independent variables. This number sets the new upper bound on the total memory capacity and thus allows it to exceed 20, as observed. These results demonstrate that expanding the reservoir’s output layer can significantly improve its computational capacity.

**Figure 7:**
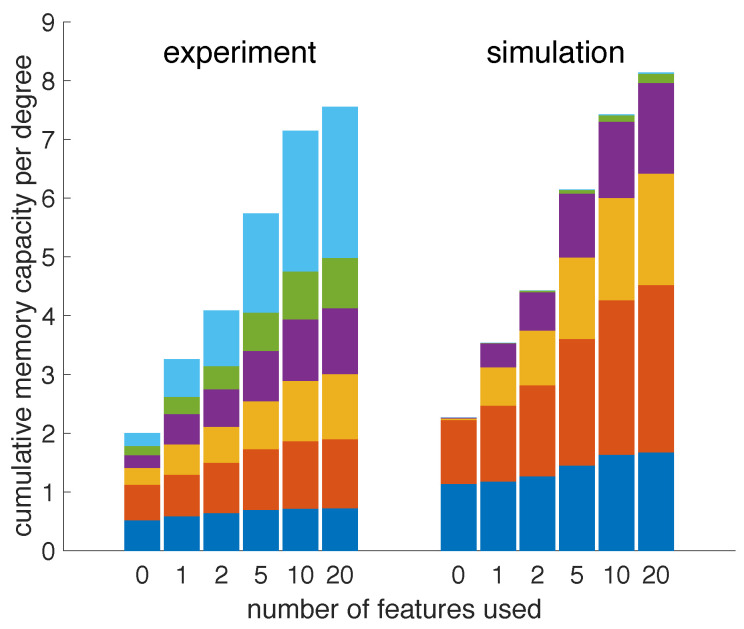
Experimental and simulated memory capacity of the reservoir computer when the number of random output features used is increased from 0 to 20. The stacked vertical bars are color-coded to represent (from the bottom up) the total memory capacities of degrees 1 (dark blue), 2 (red), 3 (orange), 4 (purple) and 5 (green) and all higher degrees combined (light blue). As such, the total height represents the total memory capacity of the system.

We have then performed both simulations and experiments with varying cavity phase. Indeed, as shown in Figure 7, both the simulated and experimental results show a significant increase in the reservoir’s total memory capacity as we increase the number of random features that are used to tune the memory-task-related readout weights. Not using any features to tune the readout weights’ case corresponds with the standard reservoir output scheme and yields the lowest total memory capacity of approximately 2. This is considerably lower than the theoretical upper bound. The discrepancy demonstrates the performance degradation caused by the phase fluctuations. When all 20 features are used in the expanded output, the memory capacity increases to approximately 8.

Both the simulated and experimental results show a smooth transition as we increase the number of features used to tune the readout weights of the memory tasks. The growing total memory capacity seems to show a linear trend, but the results are not plotted on a linear horizontal axis. On closer observation, it becomes apparent that a larger number of features yields diminishing returns. In fact, more than half of the achievable increase in total memory capacity can be obtained with as few as five random features. These results demonstrate that the presented output expansion can be exploited to partially recover the computational capacity of a reservoir affected by uncontrolled parameter variations.

Given the diminishing returns on increasing the number of random features used in the output expansion, the feature-based output expansion presented in this work allows for a powerful trade-off between system complexity and performance gains. This scalability is especially relevant to practical implementations, where the system size and the number of parameters (readout weights) that have to be optimized for task-solving are typically constrained.

In the experiment, we observe a larger contribution of the system’s nonlinear memory capacity to its overall memory capacity. We have previously demonstrated [12] that this setup’s linear and nonlinear memory capacity is very sensitive to operational parameters (such as $\beta_{0}$, for example), whereas the total memory capacity is largely unaffected. Because longer experimental runs were required for the topic at hand, the results can be affected by fluctuations in other experimental parameters not accounted for in the simulation. This could explain the discrepancy between simulated and experimental relative contributions of linear and nonlinear memory capacities.

### 3.3. Nonlinear Channel Equalization Task

In this section, we apply the same expanded output to improve the reservoir’s performance on a benchmark test inspired by telecommunications. This four-level channel equalization task, first introduced to the reservoir computing community in [2], consists of equalizing a noisy and nonlinear communication channel. The transmitted signal has the form d(n)∈{−3,−1,1,3} and propagates through a communication channel whose output u(n) is modeled by

(27) $$\begin{array}{cc} q(n)= & 0.08d(n+2)-0.12d(n+1)+d(n)+0.18d(n-1)\\ \; & -0.1d(n-2)+0.091d(n-3)-0.05d(n-4)\\ \; & +0.04d(n-5)+0.03d(n-6)+0.01d(n+7) \end{array}$$

(28) $$\begin{array}{cc} u(n)= & 0.036q^{2}\left(n\right)-0.011q^{3}\left(n\right)+\nu \left(n\right) \end{array}$$

where ν(n) is Gaussian white noise, scaled to achieve a signal to noise ratio of 32 dB. The signal u(n) serves as the input signal to the reservoir which is then trained to reconstruct d(n). We have used increasing subsets of extracted features to tune the weights for the reservoir output involved in solving this task. Due to the digital nature of the four-level target signal, the symbol error rate (SER) is used to quantify the reservoir’s performance on this task.

We have first evaluated the system’s performance when the cavity phase is stable. We have performed several experiments without active cavity stabilization and have selected a small subset of experiments with very few fluctuations of the cavity phase. For these experiments, an average SER of 3.7% is observed using the standard reservoir output (without expansion). In simulations with a stable phase, however, symbol error rates below 0.1% are observed with the standard RC output scheme. The difference between the simulated and experimental results suggests that the selected subset of experiments (with 3.7% SER) is not entirely free of phase fluctuations. When averaging over all experiments, a higher SER of 4.4% is reported (without output expansion). We can thus conclude that the experimental phase fluctuations cause an increase in the SER of at least 0.7%.

**Figure 8:**
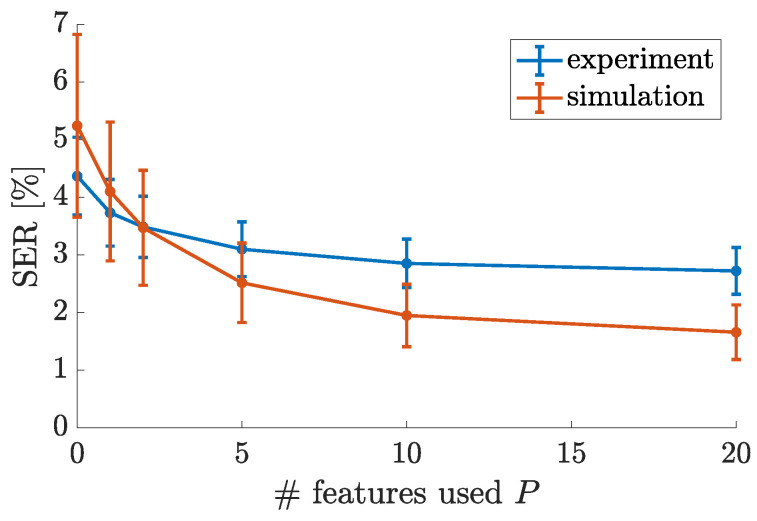
Experimental and simulated results on the 4-level channel equalization benchmark task. The symbol error rate is reported as a function of the number of random features that are used to tune the task-related readout weights. Error bars are obtained by running experiments/simulations for several iterations and using different sets of random weights for the construction of the random features.

We have then investigated the ability to recover lost performance by expanding the reservoir’s output layer using random auxiliary features. The experimental and simulated results are shown in Figure 8. We observe that the experimental SER decreases smoothly from 4.4 to 2.7% and the simulated SER decreases smoothly from 5.2 to 1.7% when the number of features used for weight-tuning is increased from 0 to 20. As these results are plotted on a linear horizontal axis, it can be readily seen that a larger number of features yields diminishing returns. Both experimental and simulated results show the same trend, although the performance gains are smaller in the experiment compared to the simulation. Interestingly the experimental and simulated results cross over, which could be explained using the memory capacities obtained in Section 3.2. The experimental system has a larger nonlinear computational capacity, which is why it outperforms the simulated systems without feature-based weight-tuning. Naturally, then, it also gains less from the nonlinearity introduced by the feature-based weight-tuning scheme. By the same reasoning, the simulated system which owes most of its nonlinear memory capacity to the weight-tuning scheme sees more significant performance gains.

We have then investigated what happens when the reservoir’s output is expanded through a (full-rank) polynomial expansion of the same degree (i.e., 2) as our feature-based approach, as outlined in the paragraph discussing Equation (10). In this case, an SER as low as 1.8% is observed in the simulation and 2.8% in the experiment. As expected, these symbol error rates are comparable to the results obtained when using all 20 random features in the feature-based output expansion.

Considering all experimental results, we have shown that the output expansion can improve the SER of a perturbed system (4.4%) to below (2.7%) the average SER of the most stable subset of experiments (3.7%). Since this subset does not perfectly represent constant system parameters, we may have overestimated the SER of a perfectly stable experimental system. The ensemble of all simulation results indeed suggests that the output expansion can bring the SER of a perturbed system (5.2%) significantly closer (1.7%) to that of an unperturbed system (<0.1%) but not surpass it.

We conclude that our example of a reservoir output expansion allows for the recovery, at least partially, of performance lost due to uncontrolled parameter variations. We also observe the same trend of diminishing returns on increasing the number of random features used in the output expansion, as was observed when evaluating the reservoir’s computational capacity.

**Figure 9:**
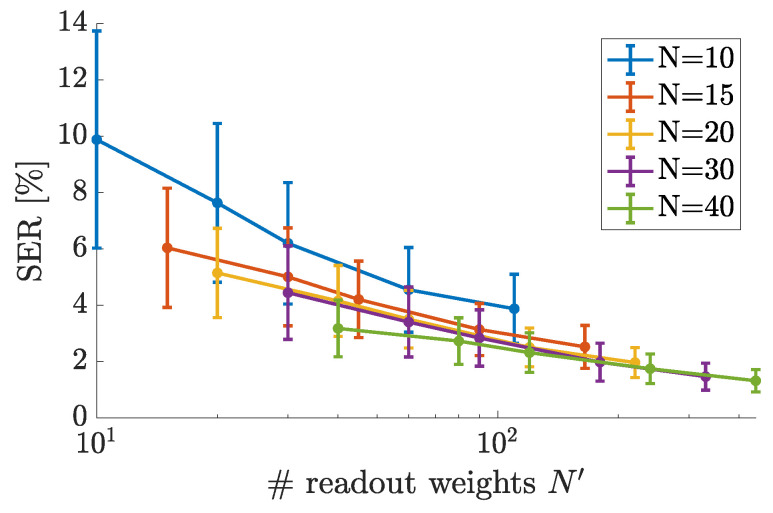
Additional simulated results on the 4-level channel equalization benchmark task. The symbol error rate is reported as a function of the total number of readout weights $N^{\prime }$ that must be optimized. Different curves represent reservoirs with different numbers of neurons *N*, ranging from 10 to 40. For each system, the number *P* of random features that are used in the output expansion is varied as 0, 1, 2, 5 and 10 from left to right, which affects $N^{\prime }=N\left(P+1\right)$. Error bars are obtained by running experiments/simulations for several iterations and using different sets of random weights for the construction of the random features.

Finally, we have investigated in simulation how the proposed output expansion affects systems with different numbers of neurons *N*. In Figure 9, we plot the SER for systems with *N* ranging from 10 to 40, noting that N=20 in all previously shown results. For a fair comparison, the horizontal axis shows the total number of readout weights $N^{\prime }$ that must be optimized (on a logarithmic scale). For all systems, we vary the number of random features *P* used in the output expansion as 0, 1, 2, 5 and 10 from left to right. The number of readout weights is affected as $N^{\prime }=N\left(P+1\right)$.

The results from Figure 9 indicate that expanding the readout layer as proposed helps improve performance for all reservoir sizes (*N*) considered, with consistent scaling of the performance versus the increase in complexity ($N^{\prime }$). However, it is not as efficient as expanding the reservoir itself. The proposed output expansion should thus be of particular use for experimental systems where expanding the output is easier than expanding the reservoir itself.

The variations in reservoir computing performance for different sets of random weights $W_{R}$ are captured by the error bars in Figure 8 and Figure 9. The observed variations are smaller than the performance gains when the system’s output is expanded with at least five random features. In that case, random feature weights $W_{R}$ can thus be expected to improve performance with great confidence.

## 4. Conclusions

We have investigated an unsupervised and scalable method to expand a reservoir’s output layer, and we have successfully exploited it to deal with slow and uncontrolled parameter variations that perturb the operation of a reservoir computer, thereby negatively affecting its computational capacity. This investigation was performed on a delay-based photonic reservoir computer built around an all-fiber cavity. An output expansion was proposed where, as an example, the set of output features was expanded through polynomial functions (of degree 1 and 2) of the neural responses. In the spirit of reservoir computing, this was implemented by mixing the neural responses with various numbers of random auxiliary features, which themselves are untrained combinations of neural responses.

We found that the random auxiliary features contain information about the drifting parameter and can be used to expand the set of output features on which a reservoir relies to construct its task-solving outputs. We have shown that large performance gains can be achieved with just a small set of auxiliary random features. Both our numerical and experimental results showed that the negative performance impact due to the parameter drifts can at least partially be mitigated through this feature-based output expansion. We accredit the performance gains/recovery to both the additional output complexity and the feature’s capacity to capture information about the uncontrolled parameter variations.

Compared to previous work on this topic, we have presented the first experimental demonstration of the feature-based output expansion on a coherent photonic reservoir and also introduced non-supervised methods for the construction of the auxiliary features. Choosing the number of features allows for the exploitation of a smooth and quasi-continuous trade-off between system complexity and RC performance gains, all without complicating the training procedure. Both the application of non-supervised methods and the scalability of the system’s size and complexity offer clear advantages over our previous work and can aid with the design of physical implementations. Although our work only handles variations of a single parameter in a very specific photonic system, the proposed scheme can, in principle, also be applied to multiple independent drifting parameters and to other types of systems based on different hardware platforms.

## Data Availability

Not applicable.

## Appendix A. Analysis of Phase-Drift in Simple Delay-Based Reservoir Computer

In this section we consider a simplified model describing a coherently driven delay-based passive linear cavity with ring topology to investigate how slow variations of the detuning affect the cavity dynamics and the neural responses. This helps shed light on how this information may be extracted from the responses. Following [9], the state update equation for the *k*-th neuron is given by

(A1) $$\begin{array}{c} X_{k}\left(n+1\right)=\alpha e^{i\phi (n)}X_{k-1}\left(n\right)+m_{k}u\left(n\right)+b_{k} \end{array}$$

with cavity roundtrip loss α, cavity detuning ϕ(n), real-valued input mask values $m_{k}$ and bias mask values $b_{k}$. Note that this neuron index *k* should be evaluated modulo *N* due to the ring topology of this delay-based reservoir. The real input data u(n) are zero mean and unit covariance

(A2) $$\begin{array}{cc} {\langle u\left(n\right)\rangle }_{n} & =0\\ {\langle u\left(n\right)u\left(n-r\right)\rangle }_{n} & =\delta_{r} \end{array}$$

using Kronecker delta notation. Because the reservoir described by Equation (A1) is linear, one can determine the general form of the dependence on slow phase variations ϕ(n). We start by applying this state update equation recursively through the neuron index *k*, using Equation (A1) to replace $X_{k-1}\left(n\right)$ for an expression containing $X_{k-2}\left(n-1\right)$, and so on. We obtain

(A3) $$\begin{array}{c} X_{k}\left(n+1\right)=\sum_{r=0}^{\infty }\alpha^{r}e^{i\sum_{s=1}^{r}\phi \left(n-s\right)}\left(m_{k-r}u\left(n-1-r\right)+b_{k-r}\right). \end{array}$$

Assuming all phase variations occur slowly with respect to variations in the input data, we can consider a time interval large enough such that Equation (A2) is satisfied, yet small enough such that we can approximate

(A4) $$\begin{array}{c} \alpha^{r}e^{i\sum_{s=1}^{r}\phi \left(n-s\right)}={\left(\alpha e^{i{\langle \phi \rangle }_{n}}\right)}^{r} \end{array}$$

with ${\langle \phi \rangle }_{n}$ the average value of quasi-constant phase ϕ(n) within the interval. From Equation (A3), it then follows that the temporal mean of the neural response filters out the input variations and retains the important phase information

(A5) $$\begin{array}{c} {\langle X_{k}\rangle }_{n}=\sum_{r=0}^{\infty }b_{k-r}{\left(\alpha e^{i{\langle \phi \rangle }_{n}}\right)}^{r}. \end{array}$$

In the case of a quadratic readout (i.e., a photodectector), we similarly find

(A6) $$\begin{array}{cc} \langle |X_{k}{{|}^{2}\rangle }_{n}= & \sum_{r=0}^{\infty }\eta_{kr}\cos\left(r{\langle \phi \rangle }_{n}\right) \end{array}$$

with constants

(A7) $$\begin{array}{c} \eta_{kr}=\left\{\begin{array}{cc} \sum_{s=0}^{\infty }|\alpha^{s}b_{k-s}{|}^{2}+\sum_{s=0}^{\infty }{\left|\alpha^{s}m_{k-s}\right|}^{2} & r=0\\ \sum_{s=r}^{\infty }2b_{k-s}b_{k-s+r}\alpha^{2s-r} & r>0. \end{array}\right. \end{array}$$

This analysis shows that, owing to the presence of an input bias $b_{k}$, the reservoir can exploit its fading memory property to access different combinations of the functions $\cos\left(r{\langle \phi \rangle }_{n}\right)$ with positive integer *r*. The slowest (non-trivial) feature that can be extracted has r=1 and could probably be constructed by combining the neural responses with weights $w_{k}^{slow}$, such that $\sum_{k=1}^{N}w_{k}^{slow}\eta_{kr}$ is maximized for r=1 and minimized for r≠1.

## Appendix B. Construction of Slow Features

Besides the use of random features, another meaningful way to construct the auxiliary features is to exploit an autonomous feature extraction process called slow feature analysis (SFA) [26]. As the name suggests, this process focuses on extracting signals with slow temporal profiles. Under the assumption that all parameter variations occur slowly with respect to the rate of change in all input data, these slow features should thus primarily contain information about the drifting parameters. Slow feature analysis constitutes a procedure to minimize the temporal average of each individual feature’s temporal derivatives under a small set of constraints to eliminate constant features as trivial solutions. Although more advanced versions of SFA exist, compatibility with the proposed readout scheme requires the following three step version. Step 1: the neural states are normalized, i.e., shifted to zero mean and scaled to unit variance. Step 2: the normalized data are sphered. This means that principal component analysis (PCA) is applied to transform the *N* normalized neural signals into *N* linear combinations which are mutually orthogonal. Additionally these signals are rescaled to have unit covariance. Step 3: PCA is applied to the temporal derivatives of the sphered data. The resulting eigenvalues contain information about temporal variations in the sphered data, where lower means slower. The resulting eigenvectors are effectively the sets of weights which we can use to combine the sphered data into slow features. By selecting the eigenvectors with the lowest corresponding eigenvalues, the slowest features are extracted. To capture the slow parameter variations, one would have to focus on the slowest extracted features, which are found in the subset of extracted features with the lowest corresponding eigenvalues in Step 3 of the extraction process. We have found that the slow features extracted using this simple but compatible version of SFA also yield RC performance gains, but this technique never outperformed the random features, which are easier to construct.
